# *trans*-(Ethene-1,2-di­yl)bis­(diphenyl­phosphine selenide)

**DOI:** 10.1107/S160053681105183X

**Published:** 2011-12-07

**Authors:** Zanele Phasha, Sizwe Makhoba, Alfred Muller

**Affiliations:** a Research Center for Synthesis and Catalysis, Department of Chemistry, University of Johannesburg (APK Campus), PO Box 524, Auckland Park, Johannesburg 2006, South Africa

## Abstract

In the title mol­ecule, C_26_H_22_P_2_Se_2_, both P atoms have distorted tetra­hedral environments, resulting in effective cone angles of 177 and 174°. Inversion twinning was detected and refined to a ratio of 0.35:0.65. Weak inter­molecular C—H⋯Se inter­actions are observed.

## Related literature

For background to the steric and electronic effects of group 15 ligands, see: Roodt *et al.* (2003 ▶); Muller *et al.* (2008 ▶). For information on cone angles, see: Tolman (1977 ▶); Otto (2001 ▶).
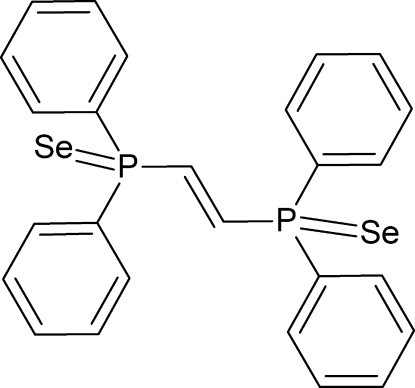


## Experimental

### 

#### Crystal data

C_26_H_22_P_2_Se_2_
*M* *_r_* = 554.3
Orthorhombic, 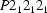

*a* = 9.0604 (9) Å
*b* = 14.3239 (14) Å
*c* = 17.9617 (18) Å
*V* = 2331.1 (4) Å^3^
*Z* = 4
Mo *K*α radiation
μ = 3.32 mm^−1^
*T* = 100 K
0.26 × 0.21 × 0.04 mm

#### Data collection

Bruker APEX DUO 4K CCD diffractometer
Absorption correction: multi-scan (*SADABS*; Bruker, 2008 ▶) *T*_min_ = 0.479, *T*_max_ = 0.879
14567 measured reflections
5814 independent reflections
5356 reflections with *I* > 2σ(*I*)
*R*_int_ = 0.028

#### Refinement

*R*[*F* ^2^ > 2σ(*F* ^2^)] = 0.025
*wR*(*F* ^2^) = 0.053
*S* = 1.01
5814 reflections
272 parameters
H-atom parameters constrained
Δρ_max_ = 0.51 e Å^−3^
Δρ_min_ = −0.48 e Å^−3^
Absolute structure: Flack (1983 ▶), 2517 Friedel pairs
Flack parameter: 0.354 (6)

### 

Data collection: *APEX2* (Bruker, 2011 ▶); cell refinement: *SAINT* (Bruker, 2008 ▶); data reduction: *SAINT* and *XPREP* (Bruker, 2008 ▶); program(s) used to solve structure: *SIR97* (Altomare *et al.*, 1999 ▶); program(s) used to refine structure: *SHELXL97* (Sheldrick, 2008 ▶); molecular graphics: *DIAMOND* (Brandenburg & Putz, 2005 ▶); software used to prepare material for publication: *WinGX* (Farrugia, 1999 ▶).

## Supplementary Material

Crystal structure: contains datablock(s) global, I. DOI: 10.1107/S160053681105183X/fj2485sup1.cif

Structure factors: contains datablock(s) I. DOI: 10.1107/S160053681105183X/fj2485Isup2.hkl

Additional supplementary materials:  crystallographic information; 3D view; checkCIF report

## Figures and Tables

**Table 1:** Hydrogen-bond geometry (Å, °)

*D*—H⋯*A*	*D*—H	H⋯*A*	*D*⋯*A*	*D*—H⋯*A*
C3—H3⋯Se2^i^	0.95	3.02	3.953 (2)	168
C21—H21⋯Se1^ii^	0.95	3.06	3.812 (2)	138
C17—H17⋯Se2^iii^	0.95	3.01	3.885 (3)	155

Symmetry codes: (i) 
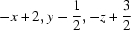
; (ii) 
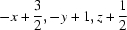
; (iii) 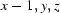.

**Fig. 1.**
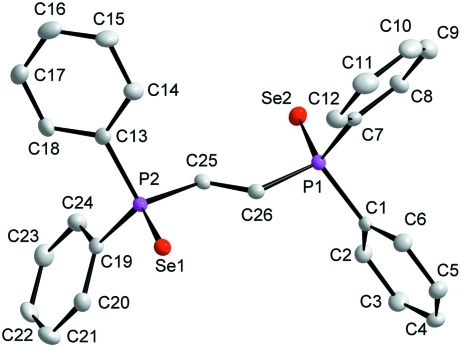
: View of the title compound indicating labelling and displacement ellipsoids (drawn at a 50% probability level).

**Fig. 2.**
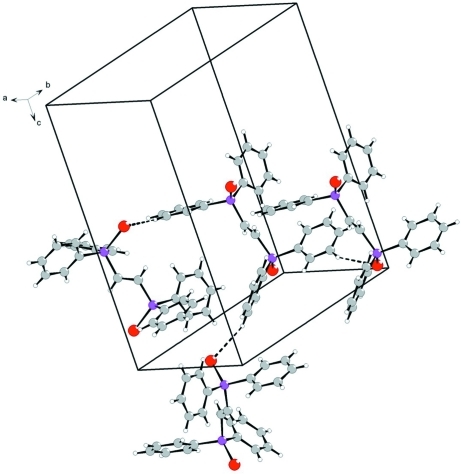
: Partial packing diagram of the title compound illustrating the weak C–H···Se interactions stabilizing the crystal packing.

## supplementary crystallographic information

### Comment

The study of the transition metal phosphorous bond spans over several decades using various techniques such as crystallography, multi nuclear NMR and IR (Roodt *et al.*, 2003). As part of this systematic investigation we have extended this study to selenium derivatives of the phosphorus ligands (see Muller *et al.*, 2008). Reported as part of the above continuing study, the single-crystal structure of the bis-phosphorus containing compound, (SePPh_2_)_2_C_2_H_2_ where Ph = C_6_H_5_, is reported here.

The structure of the title compound (see Figure 1, Table 1) shows distorted tetrahedral environments for both the phosphorus centers. The P═Se bond distances (2.1026 (6), 2.1054 (6) Å for Se1 and Se2 respectively) are marginally statistically different, possibly due to the weak C—H···Se intermolecular hydrogen bonding observed (see Figure 2, Table 2; comparison based on 1% normal distribution coefficient).

The phosphorus ligand bulkiness was evaluated by using an adaptation of the well known Tolman cone angle model (Tolman, 1977). Instead of using a CPK model, the actual geometry from the crystal structure was taken to determine an 'effective cone angle' (Otto *et al.* 2001). The Se═P distances were also adjusted to 2.28 Å (the default value used by Tolman) to remove the effect of bond distance variation. Two different cone angles of 177° and 174° were obtained for P_1_and P_2_ respectively. The difference in cone angles may also be attributed to the weak interactions mentioned previously.

### Experimental

*Trans*-1,2-bis(diphenylphosphino)ethylene and KSeCN were purchased from Sigma-Aldrich and used without purification. Eqimolar amounts of KSeCN and the *trans*-1,2-bis(diphenylphosphino)ethylene compound (*ca* 0.04 mmol) were dissolved in the minimum amounts of methanol (10 - 20 ml). The KSeCN solution was added drop wise (5 min.) to the phosphine solution with stirring at room temperature. The final solution was left to evaporate slowly until dry to give crystals suitable for a single-crystal X-ray study.

### Refinement

All hydrogen atoms were positioned in geometrically idealized positions with C—H = 0.95 Å and allowed to ride on their parent atoms with *U*_iso_(H) = 1.2*U*_eq_. The Flack parameter (based on 2517 Friedel pairs) indicates racemic twinning of the compound. This refined to a 35.4:64.6 racemic twin. The highest residual electron density of 0.51 e.Å^-3^ is 0.98 Å from Se1 representing no physical meaning.

### Crystal data

**Table:**

C_26_H_22_P_2_Se_2_	*F*(000) = 1104
*M**_r_* = 554.3	*D*_x_ = 1.579 Mg m^−^^3^
Orthorhombic, *P*2_1_2_1_2_1_	Mo *K*α radiation, λ = 0.71073 Å
Hall symbol: P 2ac 2ab	Cell parameters from 5956 reflections
*a* = 9.0604 (9) Å	θ = 2.3–28.2°
*b* = 14.3239 (14) Å	µ = 3.32 mm^−^^1^
*c* = 17.9617 (18) Å	*T* = 100 K
*V* = 2331.1 (4) Å^3^	Plate, colourless
*Z* = 4	0.26 × 0.21 × 0.04 mm

### Data collection

**Table:**

Bruker APEX DUO 4K CCD diffractometer	5814 independent reflections
graphite	5356 reflections with *I* > 2σ(*I*)
Detector resolution: 8.4 pixels mm^-1^	*R*_int_ = 0.028
φ and ω scans	θ_max_ = 28.5°, θ_min_ = 1.8°
Absorption correction: multi-scan (*SADABS*; Bruker, 2008)	*h* = −10→12
*T*_min_ = 0.479, *T*_max_ = 0.879	*k* = −12→19
14567 measured reflections	*l* = −24→22

### Refinement

**Table:**

Refinement on *F*^2^	Secondary atom site location: difference Fourier map
Least-squares matrix: full	Hydrogen site location: inferred from neighbouring sites
*R*[*F*^2^ > 2σ(*F*^2^)] = 0.025	H-atom parameters constrained
*wR*(*F*^2^) = 0.053	*w* = 1/[σ^2^(*F*_o_^2^) + (0.0268*P*)^2^] where *P* = (*F*_o_^2^ + 2*F*_c_^2^)/3
*S* = 1.01	(Δ/σ)_max_ = 0.001
5814 reflections	Δρ_max_ = 0.51 e Å^−^^3^
272 parameters	Δρ_min_ = −0.48 e Å^−^^3^
0 restraints	Absolute structure: Flack (1983), 2517 Friedel pairs
Primary atom site location: structure-invariant direct methods	Flack parameter: 0.354 (6)

### Special details

**Table:**

Experimental. The intensity data was collected on a Bruker Apex DUO 4 K CCD diffractometer using an exposure time of 20 s/frame. A total of 588 frames were collected with a frame width of 0.5° covering up to θ = 28.49° with 99.2% completeness accomplished.Analytical data: ^1^H NMR (CDCl_3_, 400 MHz) δ 7.50–7.70 (m, 10H), 7.51–7.42 (m, 10H), 7.89 (t, ^3^*J* = 22.8 Hz, 2H); ^13^C {H} NMR (CDCl_3_, 100 MHz) δ 142.4 (ethylene), 132.1, 131.8, 129.0 (Ar); ^31^P {H} NMR (CDCl_3_, 160 MHz):δ = 28.58 (dd, ^1^*J*_Se—P_ = 694.9, 814.8 Hz, 2P).
Geometry. All s.u.'s (except the s.u. in the dihedral angle between two l.s. planes) are estimated using the full covariance matrix. The cell s.u.'s are taken into account individually in the estimation of s.u.'s in distances, angles and torsion angles; correlations between s.u.'s in cell parameters are only used when they are defined by crystal symmetry. An approximate (isotropic) treatment of cell s.u.'s is used for estimating s.u.'s involving l.s. planes.
Refinement. Refinement of *F*^2^ against ALL reflections. The weighted *R*-factor *wR* and goodness of fit *S* are based on *F*^2^, conventional *R*-factors *R* are based on *F*, with *F* set to zero for negative *F*^2^. The threshold expression of *F*^2^ > 2σ(*F*^2^) is used only for calculating *R*-factors(gt) *etc*. and is not relevant to the choice of reflections for refinement. *R*-factors based on *F*^2^ are statistically about twice as large as those based on *F*, and *R*- factors based on ALL data will be even larger.

### Fractional atomic coordinates and isotropic or equivalent isotropic displacement parameters (Å^2^)

**Table:**

	*x*	*y*	*z*	*U*_iso_*/*U*_eq_	
C1	1.0685 (2)	0.72411 (15)	0.64399 (13)	0.0134 (4)	
C2	1.0826 (3)	0.62874 (15)	0.62917 (14)	0.0185 (5)	
H2	0.9975	0.5921	0.619	0.022*	
C3	1.2215 (3)	0.58774 (15)	0.62938 (15)	0.0226 (5)	
H3	1.231	0.5228	0.6198	0.027*	
C4	1.3460 (3)	0.64076 (16)	0.64339 (15)	0.0197 (5)	
H4	1.4407	0.6123	0.6434	0.024*	
C5	1.3321 (3)	0.73551 (16)	0.65749 (14)	0.0186 (5)	
H5	1.4175	0.7721	0.6668	0.022*	
C6	1.1940 (3)	0.77686 (15)	0.65806 (13)	0.0173 (5)	
H6	1.185	0.8417	0.6681	0.021*	
C7	0.9158 (2)	0.89938 (15)	0.62504 (14)	0.0158 (5)	
C8	0.9380 (3)	0.92399 (16)	0.55117 (14)	0.0198 (5)	
H8	0.939	0.8771	0.5138	0.024*	
C9	0.9588 (3)	1.01653 (18)	0.53168 (17)	0.0265 (6)	
H9	0.9743	1.033	0.481	0.032*	
C10	0.9571 (3)	1.08522 (17)	0.58612 (17)	0.0278 (6)	
H10	0.9707	1.1488	0.5726	0.033*	
C11	0.9357 (3)	1.06180 (17)	0.65944 (18)	0.0282 (6)	
H11	0.935	1.1091	0.6965	0.034*	
C12	0.9150 (3)	0.96844 (16)	0.67974 (16)	0.0221 (5)	
H12	0.9004	0.9522	0.7305	0.027*	
C13	0.4690 (2)	0.81404 (15)	0.84948 (14)	0.0156 (5)	
C14	0.4717 (3)	0.90052 (16)	0.81243 (15)	0.0212 (5)	
H14	0.5622	0.9242	0.7935	0.025*	
C15	0.3430 (3)	0.95120 (17)	0.80341 (16)	0.0252 (6)	
H15	0.3451	1.0095	0.7782	0.03*	
C16	0.2105 (3)	0.91681 (17)	0.83126 (15)	0.0250 (6)	
H16	0.1221	0.9516	0.825	0.03*	
C17	0.2075 (3)	0.83228 (17)	0.86790 (15)	0.0244 (5)	
H17	0.1169	0.8088	0.8869	0.029*	
C18	0.3369 (3)	0.78136 (17)	0.87717 (14)	0.0211 (5)	
H18	0.3343	0.7234	0.9029	0.025*	
C19	0.5966 (2)	0.63390 (15)	0.88948 (14)	0.0145 (5)	
C20	0.6670 (3)	0.59705 (15)	0.95104 (14)	0.0192 (5)	
H20	0.737	0.6336	0.9775	0.023*	
C21	0.6355 (3)	0.50619 (16)	0.97447 (16)	0.0245 (6)	
H21	0.6852	0.4806	1.0164	0.029*	
C22	0.5324 (3)	0.45375 (16)	0.93677 (16)	0.0230 (6)	
H22	0.5092	0.3925	0.9534	0.028*	
C23	0.4621 (3)	0.49016 (17)	0.87437 (17)	0.0243 (6)	
H23	0.3923	0.4535	0.8479	0.029*	
C24	0.4944 (3)	0.58029 (17)	0.85093 (16)	0.0214 (5)	
H24	0.4464	0.6053	0.8084	0.026*	
C25	0.7048 (2)	0.74177 (14)	0.76513 (12)	0.0142 (4)	
H25	0.6431	0.711	0.7301	0.017*	
C26	0.8328 (2)	0.77552 (14)	0.74213 (13)	0.0147 (4)	
H26	0.8992	0.8002	0.778	0.018*	
P1	0.88646 (6)	0.77673 (4)	0.64539 (3)	0.01278 (11)	
P2	0.64250 (6)	0.75184 (4)	0.86038 (3)	0.01338 (12)	
Se1	0.73133 (2)	0.712573 (15)	0.574954 (15)	0.01794 (6)	
Se2	0.79614 (2)	0.821665 (14)	0.928447 (14)	0.01582 (5)	

### Atomic displacement parameters (Å^2^)

**Table:**

	*U*^11^	*U*^22^	*U*^33^	*U*^12^	*U*^13^	*U*^23^
C1	0.0146 (10)	0.0147 (10)	0.0108 (10)	0.0023 (8)	0.0025 (9)	0.0012 (8)
C2	0.0186 (11)	0.0144 (11)	0.0226 (14)	−0.0001 (9)	0.0022 (11)	−0.0013 (9)
C3	0.0248 (12)	0.0135 (11)	0.0295 (14)	0.0039 (9)	0.0034 (12)	−0.0008 (9)
C4	0.0178 (11)	0.0191 (11)	0.0224 (13)	0.0056 (9)	−0.0008 (11)	0.0032 (10)
C5	0.0155 (11)	0.0190 (11)	0.0213 (13)	−0.0012 (9)	−0.0027 (10)	−0.0005 (9)
C6	0.0200 (11)	0.0127 (10)	0.0193 (12)	0.0014 (9)	0.0001 (10)	−0.0049 (9)
C7	0.0123 (10)	0.0174 (11)	0.0178 (13)	0.0019 (8)	−0.0017 (10)	0.0017 (9)
C8	0.0250 (12)	0.0168 (11)	0.0176 (13)	0.0007 (10)	−0.0004 (11)	0.0001 (9)
C9	0.0265 (14)	0.0257 (14)	0.0274 (16)	−0.0004 (11)	0.0010 (13)	0.0078 (11)
C10	0.0263 (12)	0.0147 (12)	0.0423 (19)	−0.0005 (9)	−0.0028 (14)	0.0064 (11)
C11	0.0316 (14)	0.0149 (12)	0.0379 (18)	0.0000 (11)	−0.0067 (14)	−0.0048 (11)
C12	0.0249 (12)	0.0184 (11)	0.0230 (14)	0.0000 (10)	−0.0028 (12)	−0.0010 (10)
C13	0.0182 (10)	0.0151 (11)	0.0136 (11)	0.0030 (9)	0.0024 (10)	−0.0028 (9)
C14	0.0209 (12)	0.0174 (12)	0.0252 (14)	−0.0010 (9)	−0.0023 (11)	0.0023 (10)
C15	0.0269 (13)	0.0172 (11)	0.0317 (16)	0.0059 (10)	−0.0039 (12)	0.0034 (10)
C16	0.0239 (12)	0.0254 (12)	0.0259 (14)	0.0100 (11)	−0.0037 (12)	−0.0062 (10)
C17	0.0193 (11)	0.0306 (13)	0.0232 (14)	0.0027 (11)	0.0034 (12)	0.0003 (10)
C18	0.0241 (12)	0.0227 (12)	0.0165 (12)	0.0032 (10)	0.0061 (11)	0.0039 (10)
C19	0.0153 (10)	0.0114 (11)	0.0168 (12)	0.0026 (8)	0.0044 (10)	−0.0009 (9)
C20	0.0184 (11)	0.0187 (11)	0.0206 (13)	−0.0023 (9)	0.0001 (10)	0.0001 (9)
C21	0.0328 (14)	0.0183 (12)	0.0225 (15)	0.0018 (11)	−0.0029 (12)	0.0048 (10)
C22	0.0283 (12)	0.0149 (11)	0.0257 (15)	−0.0027 (9)	0.0049 (12)	0.0054 (11)
C23	0.0233 (12)	0.0208 (12)	0.0288 (16)	−0.0062 (10)	0.0010 (12)	−0.0026 (11)
C24	0.0216 (12)	0.0219 (12)	0.0207 (13)	−0.0009 (9)	−0.0024 (11)	0.0029 (10)
C25	0.0189 (10)	0.0106 (9)	0.0130 (11)	0.0014 (9)	0.0008 (10)	0.0000 (8)
C26	0.0165 (10)	0.0135 (10)	0.0140 (11)	0.0019 (8)	−0.0014 (9)	−0.0001 (8)
P1	0.0135 (2)	0.0126 (3)	0.0122 (3)	0.0001 (2)	0.0011 (2)	−0.0005 (2)
P2	0.0148 (3)	0.0124 (3)	0.0130 (3)	0.0017 (2)	0.0019 (2)	0.0009 (2)
Se1	0.01645 (10)	0.01938 (11)	0.01798 (12)	−0.00169 (8)	−0.00126 (11)	−0.00443 (9)
Se2	0.01802 (10)	0.01372 (10)	0.01572 (11)	0.00030 (8)	0.00040 (11)	−0.00065 (9)

### Geometric parameters (Å, °)

**Table:**

C1—C6	1.388 (3)	C14—H14	0.95
C1—C2	1.398 (3)	C15—C16	1.391 (4)
C1—P1	1.813 (2)	C15—H15	0.95
C2—C3	1.389 (3)	C16—C17	1.378 (4)
C2—H2	0.95	C16—H16	0.95
C3—C4	1.383 (3)	C17—C18	1.391 (3)
C3—H3	0.95	C17—H17	0.95
C4—C5	1.386 (3)	C18—H18	0.95
C4—H4	0.95	C19—C20	1.381 (3)
C5—C6	1.384 (3)	C19—C24	1.388 (3)
C5—H5	0.95	C19—P2	1.817 (2)
C6—H6	0.95	C20—C21	1.397 (3)
C7—C8	1.387 (3)	C20—H20	0.95
C7—C12	1.394 (3)	C21—C22	1.377 (4)
C7—P1	1.814 (2)	C21—H21	0.95
C8—C9	1.384 (3)	C22—C23	1.391 (4)
C8—H8	0.95	C22—H22	0.95
C9—C10	1.387 (4)	C23—C24	1.389 (3)
C9—H9	0.95	C23—H23	0.95
C10—C11	1.373 (4)	C24—H24	0.95
C10—H10	0.95	C25—C26	1.322 (3)
C11—C12	1.399 (3)	C25—P2	1.807 (2)
C11—H11	0.95	C25—H25	0.95
C12—H12	0.95	C26—P1	1.804 (2)
C13—C18	1.377 (3)	C26—H26	0.95
C13—C14	1.406 (3)	P1—Se1	2.1026 (6)
C13—P2	1.818 (2)	P2—Se2	2.1054 (6)
C14—C15	1.383 (3)		
			
C6—C1—C2	119.4 (2)	C17—C16—C15	120.0 (2)
C6—C1—P1	121.07 (16)	C17—C16—H16	120
C2—C1—P1	119.47 (17)	C15—C16—H16	120
C3—C2—C1	119.7 (2)	C16—C17—C18	120.1 (2)
C3—C2—H2	120.1	C16—C17—H17	120
C1—C2—H2	120.1	C18—C17—H17	120
C4—C3—C2	120.5 (2)	C13—C18—C17	120.7 (2)
C4—C3—H3	119.8	C13—C18—H18	119.6
C2—C3—H3	119.8	C17—C18—H18	119.6
C3—C4—C5	119.8 (2)	C20—C19—C24	119.7 (2)
C3—C4—H4	120.1	C20—C19—P2	118.68 (18)
C5—C4—H4	120.1	C24—C19—P2	121.57 (19)
C6—C5—C4	120.1 (2)	C19—C20—C21	120.2 (2)
C6—C5—H5	119.9	C19—C20—H20	119.9
C4—C5—H5	119.9	C21—C20—H20	119.9
C5—C6—C1	120.40 (19)	C22—C21—C20	119.9 (2)
C5—C6—H6	119.8	C22—C21—H21	120
C1—C6—H6	119.8	C20—C21—H21	120
C8—C7—C12	119.6 (2)	C21—C22—C23	120.2 (2)
C8—C7—P1	117.39 (19)	C21—C22—H22	119.9
C12—C7—P1	123.0 (2)	C23—C22—H22	119.9
C9—C8—C7	120.3 (2)	C24—C23—C22	119.8 (2)
C9—C8—H8	119.8	C24—C23—H23	120.1
C7—C8—H8	119.8	C22—C23—H23	120.1
C8—C9—C10	120.0 (3)	C19—C24—C23	120.2 (3)
C8—C9—H9	120	C19—C24—H24	119.9
C10—C9—H9	120	C23—C24—H24	119.9
C11—C10—C9	120.3 (2)	C26—C25—P2	122.73 (18)
C11—C10—H10	119.8	C26—C25—H25	118.6
C9—C10—H10	119.8	P2—C25—H25	118.6
C10—C11—C12	120.2 (3)	C25—C26—P1	122.73 (18)
C10—C11—H11	119.9	C25—C26—H26	118.6
C12—C11—H11	119.9	P1—C26—H26	118.6
C7—C12—C11	119.6 (3)	C26—P1—C1	104.75 (11)
C7—C12—H12	120.2	C26—P1—C7	104.06 (11)
C11—C12—H12	120.2	C1—P1—C7	105.45 (10)
C18—C13—C14	119.0 (2)	C26—P1—Se1	113.27 (8)
C18—C13—P2	123.07 (18)	C1—P1—Se1	114.70 (8)
C14—C13—P2	117.87 (18)	C7—P1—Se1	113.58 (8)
C15—C14—C13	120.2 (2)	C25—P2—C19	105.65 (10)
C15—C14—H14	119.9	C25—P2—C13	101.97 (11)
C13—C14—H14	119.9	C19—P2—C13	106.79 (11)
C14—C15—C16	120.0 (2)	C25—P2—Se2	112.39 (8)
C14—C15—H15	120	C19—P2—Se2	115.22 (8)
C16—C15—H15	120	C13—P2—Se2	113.68 (8)
			
C6—C1—C2—C3	0.6 (4)	P2—C25—C26—P1	−173.15 (11)
P1—C1—C2—C3	−178.8 (2)	C25—C26—P1—C1	−129.02 (19)
C1—C2—C3—C4	−0.7 (4)	C25—C26—P1—C7	120.51 (19)
C2—C3—C4—C5	0.1 (4)	C25—C26—P1—Se1	−3.3 (2)
C3—C4—C5—C6	0.4 (4)	C6—C1—P1—C26	−84.4 (2)
C4—C5—C6—C1	−0.5 (4)	C2—C1—P1—C26	95.0 (2)
C2—C1—C6—C5	0.0 (4)	C6—C1—P1—C7	25.1 (2)
P1—C1—C6—C5	179.38 (19)	C2—C1—P1—C7	−155.5 (2)
C12—C7—C8—C9	−0.2 (4)	C6—C1—P1—Se1	150.79 (18)
P1—C7—C8—C9	179.00 (19)	C2—C1—P1—Se1	−29.8 (2)
C7—C8—C9—C10	−0.2 (4)	C8—C7—P1—C26	−170.97 (19)
C8—C9—C10—C11	0.4 (4)	C12—C7—P1—C26	8.2 (2)
C9—C10—C11—C12	−0.2 (4)	C8—C7—P1—C1	79.1 (2)
C8—C7—C12—C11	0.4 (4)	C12—C7—P1—C1	−101.8 (2)
P1—C7—C12—C11	−178.79 (19)	C8—C7—P1—Se1	−47.3 (2)
C10—C11—C12—C7	−0.2 (4)	C12—C7—P1—Se1	131.83 (19)
C18—C13—C14—C15	−0.7 (4)	C26—C25—P2—C19	−126.76 (19)
P2—C13—C14—C15	−179.2 (2)	C26—C25—P2—C13	121.8 (2)
C13—C14—C15—C16	0.2 (4)	C26—C25—P2—Se2	−0.3 (2)
C14—C15—C16—C17	0.1 (4)	C20—C19—P2—C25	122.47 (19)
C15—C16—C17—C18	0.1 (4)	C24—C19—P2—C25	−56.8 (2)
C14—C13—C18—C17	0.9 (4)	C20—C19—P2—C13	−129.50 (19)
P2—C13—C18—C17	179.33 (19)	C24—C19—P2—C13	51.2 (2)
C16—C17—C18—C13	−0.6 (4)	C20—C19—P2—Se2	−2.2 (2)
C24—C19—C20—C21	0.0 (4)	C24—C19—P2—Se2	178.50 (18)
P2—C19—C20—C21	−179.30 (19)	C18—C13—P2—C25	125.2 (2)
C19—C20—C21—C22	−1.0 (4)	C14—C13—P2—C25	−56.3 (2)
C20—C21—C22—C23	1.6 (4)	C18—C13—P2—C19	14.6 (3)
C21—C22—C23—C24	−1.1 (4)	C14—C13—P2—C19	−166.89 (19)
C20—C19—C24—C23	0.4 (4)	C18—C13—P2—Se2	−113.6 (2)
P2—C19—C24—C23	179.7 (2)	C14—C13—P2—Se2	64.9 (2)
C22—C23—C24—C19	0.1 (4)		

### Hydrogen-bond geometry (Å, °)

**Table:**

*D*—H···*A*	*D*—H	H···*A*	*D*···*A*	*D*—H···*A*
C3—H3···Se2^i^	0.95	3.02	3.953 (2)	168
C21—H21···Se1^ii^	0.95	3.06	3.812 (2)	138
C17—H17···Se2^iii^	0.95	3.01	3.885 (3)	155

Symmetry codes: (i) −*x*+2, *y*−1/2, −*z*+3/2; (ii) −*x*+3/2, −*y*+1, *z*+1/2; (iii) *x*−1, *y*, *z*.
